# USP14 Mediates Molecular Mechanisms Regulating Aortic Valve Stenosis Through Ubiquitination

**DOI:** 10.1111/jcmm.70765

**Published:** 2025-09-23

**Authors:** Yin Yang, Bo‐Chen Yao, Jing‐Hui Li, Qing‐Liang Chen, Nan Jiang, Lian‐Qun Wang, Zhi‐Gang Guo

**Affiliations:** ^1^ Cardiovascular Surgery Tianjin Medical University, Tianjin Chest Hospital Tianjin People's Republic of China

**Keywords:** aortic valve stenosis, CDK4, regulatory mechanism, therapeutic target, USP14

## Abstract

The incidence of aortic valve stenosis (AVS) has been increasing in recent years, making it one of the leading causes of cardiovascular‐related deaths among the elderly. Clinical samples from 30 AVS patients and 30 controls treated at Tianjin Chest Hospital between 2010 and 2020 were collected. Analyses included immunofluorescence detection, Western blotting (WB), quantitative RT‐PCR analysis (qRT‐PCR), haematoxylin and eosin (HE) staining and molecular docking experiments of protein–protein interactions. USP14 was found to be highly expressed in AVS tissues, which was validated by immunofluorescence and WB analyses. qRT‐PCR results indicated that the mRNA expression levels of USP14 and CDK4 were significantly elevated in AVS tissues. HE staining revealed significant pathological changes in AVS tissues. Molecular docking experiments demonstrated the interaction between USP14 and CDK4, suggesting a potential regulatory mechanism in AVS. USP14 may be involved in the occurrence and development of AVS by regulating cell proliferation, apoptosis and fibrosis processes. It may serve as a therapeutic target for treating AVS.

**Abbreviations**
AVS: aortic valve stenosis
HE: haematoxylin and eosin
qRT‐PCR: quantitative RT‐PCR analysis
WB: Western blotting

## 1 Introduction

Aortic valve stenosis (AVS) is a common valvular heart disease with a rising incidence in recent years. Currently, age‐related degenerative aortic valve stenosis has become the most common cause of AVS in adults [1]. The pathological processes of AVS include proliferative inflammation, lipid accumulation, activation of angiotensin‐converting enzyme, infiltration by macrophages and T lymphocytes, ultimately leading to aortic valve calcification. The calcification of the valve base restricts valve movement, resulting in aortic valve orifice narrowing. Hypertension, dyslipidaemia, diabetes and smoking are risk factors for its occurrence [2, 3]. Although AVS is a slowly progressive disease, rapid diagnosis and treatment are crucial once symptoms appear or obstruction becomes severe [4]. Current treatments for AVS include balloon valvuloplasty, surgical aortic valve replacement and transcatheter aortic valve replacement [5]. However, surgical treatments themselves may cause new tissue damage and associated complications. Despite extensive research on the clinical manifestations and hazards of AVS, its pathogenesis remains not fully understood. Existing studies indicate that genetic factors, chromosomal abnormalities and gene fusions may be closely related to the development of AVS. In order to better understand the molecular mechanisms of AVS and develop effective diagnostic and therapeutic methods, in‐depth research on related genes and molecular pathways is particularly important.

The ubiquitin‐specific protease 14 (USP14) is a deubiquitinating enzyme, unique in the USP family for its ability to associate with the proteasome. It can be recruited to the 19S particle, where it prevents substrates with ubiquitin chains from being recognised and degraded by the proteasome by cleaving these chains [6]. Accordingly, inhibition of USP14 can enhance proteasome degradation activity [7]. USP14 is amplified and overexpressed in various types of diseases [8, 9, 10, 11]. Its expression plays a pathogenic role in various diseases and is negatively correlated with disease prognosis [12]. Our research has found that USP14 is highly expressed in stenotic aortic valve tissues, but its specific role in AVS has not been thoroughly studied.

In recent years, the rapid development of bioinformatics technologies has provided powerful tools for studying the molecular mechanisms of diseases. Microarray technology can explore and identify differentially expressed genes related to diseases using genomic data [13]. Bioinformatics analysis can reveal the functions and pathways of these genes, identifying potential biomarkers and therapeutic targets, thus providing a foundation for further experimental research. Therefore, this study focuses on utilising microarray technology in preliminary work to identify differentially expressed genes between AVS and normal aortic valves, and to identify core targets. Functional experiments will then be used for further validation.

## 2 Methods

### 2.1 Study Subjects and Sample Collection

Clinical samples were collected from 30 aortic valve stenosis (AVS) patients and 30 non‐stenotic (CON) patients treated at Tianjin Chest Hospital between 2010 and 2020. These specimens were collected prior to any auxiliary treatments. All cases were confirmed by clinical, imaging and pathological diagnoses, and had complete follow‐up data. Fresh tissue samples were immediately preserved in liquid nitrogen for subsequent experiments.

### 2.2 Immunofluorescence Detection

Immunofluorescence detection was performed to assess the expression and localisation of USP14 and CDK4 proteins in AVS and CON tissues. Separate control groups with non‐stenotic aortic valves and experimental groups with stenotic aortic valves were established. After incubation with primary and secondary antibodies, the protein expression and localisation were observed using a laser confocal microscope.

### 2.3 Western Blot Analysis

Proteins were extracted from aortic valve tissues and quantified using a BCA protein assay kit. Equal amounts of protein from each sample were separated by SDS‐PAGE and transferred to PVDF membranes. The membranes were blocked with 5% non‐fat milk in TBST (Tris‐buffered saline with 0.1% Tween‐20) for 1 h at room temperature, then incubated overnight at 4°C with primary antibodies against USP14, CDK4 and β‐actin (as a loading control). After washing, the membranes were incubated with HRP‐conjugated secondary antibodies at room temperature for 1 h. Protein bands were visualised using an enhanced chemiluminescence detection system and captured on x‐ray film. Relative protein expression levels were quantified using ImageJ software.

### 2.4 Quantitative RT‐PCR Analysis

Quantitative RT‐PCR (qRT‐PCR) was used to measure the mRNA expression levels of USP14 and CDK4 in AVS and CON tissues. Total RNA was extracted and cDNA was synthesised using a reverse transcription kit. PCR amplification was performed using specific primers in a reaction system containing SYBR Green dye. Detection and data analysis were conducted using a fluorescence quantitative PCR instrument.

### 2.5 Haematoxylin and Eosin Staining

Tissue samples from stenotic and non‐stenotic aortic valves were fixed in 10% neutral buffered formalin to preserve cellular structures. Fixed tissues were embedded in paraffin and sectioned. The paraffin blocks were sliced into 4–5 μm thick sections using a microtome. Sections were mounted on slides and dried at room temperature. Slides were deparaffinised in xylene and rehydrated through graded alcohols to water. Slides were then immersed in haematoxylin solution to stain nuclei blue. After rinsing with tap water, differentiation was performed using acid alcohol (1% HCl in 70% ethanol) to remove non‐specific staining. Slides were rinsed again with running water and dipped in lithium carbonate or ammonia water to enhance nuclear blue staining. Slides were counterstained with eosin solution to stain cytoplasm and extracellular matrix pink. After rinsing to remove excess eosin, sections were dehydrated through graded alcohols to absolute alcohol. Clearing was performed in xylene, and sections were mounted with synthetic resin. Stained sections were observed and imaged under a light microscope at 100× magnification to evaluate histopathological changes, focusing on nuclear and cytoplasmic staining and tissue structural integrity.

### 2.6 Molecular Docking of Protein–Protein Interactions

#### 2.6.1 Preparation of Protein Structures

The structures of USP14 and CDK4 target proteins were obtained from the RCSB database (https://www.rcsb.org/). All protein structures were processed using the Molecular Operating Environment (MOE 2019.1) platform with the Amber10 force field, which included removing water and ions, protonation, adding missing atoms and groups and energy minimisation of proteins.

#### 2.6.2 Molecular Docking

The HDOCK server was used to predict binding complexes between two molecules, such as proteins and nucleic acids, using a hybrid docking strategy. Each protein was set as rigid, and the docking contact site was set to the full surface. After docking, 100 conformations were generated, and docking scores were calculated using the ITScorePP iterative knowledge‐based scoring function. More negative docking scores indicated more likely binding models. The conformation with the most negative score was selected and optimised using the Minimization module in the MOE 2019.1 software to address unreasonable spatial contacts in rigid docking. The energy minimisation used the Amber10.

Force field and a water solvent model, with optimisation in two steps: Steepest Descent and Conjugate Gradient, both with a maximum of 5000 iterations. The optimised results were visualised and analysed using Pymol2.1 software.

### 2.7 Statistical Analysis

Statistical analysis of experimental data was performed using SPSS 24.0 software. Results for each parameter were expressed as mean ± standard deviation (±). Statistical significance was assessed using t‐tests or one‐way analysis of variance. Differences with *p* < 0.05 were considered statistically significant, and differences with *p* < 0.01 were considered highly statistically significant.

## 3 Results

### 3.1 Western Blot Analysis

**FIGURE 1:**
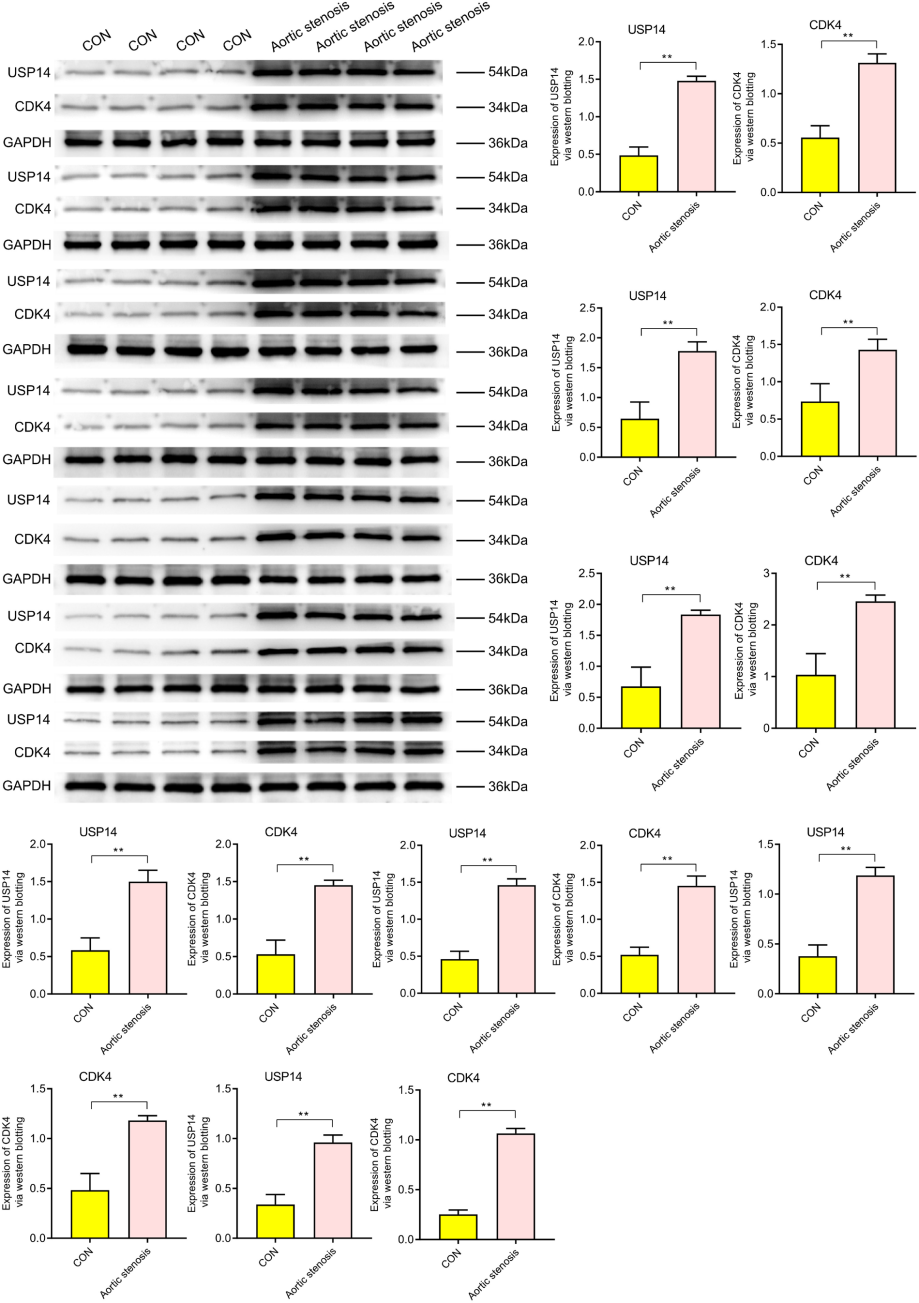
Western blot analysis of USP14 and CDK4 in aortic stenosis (AS) and control (CON) tissues. The expression of USP14 and CDK4 is significantly higher in the AS group compared to the CON group.

**FIGURE 2:**
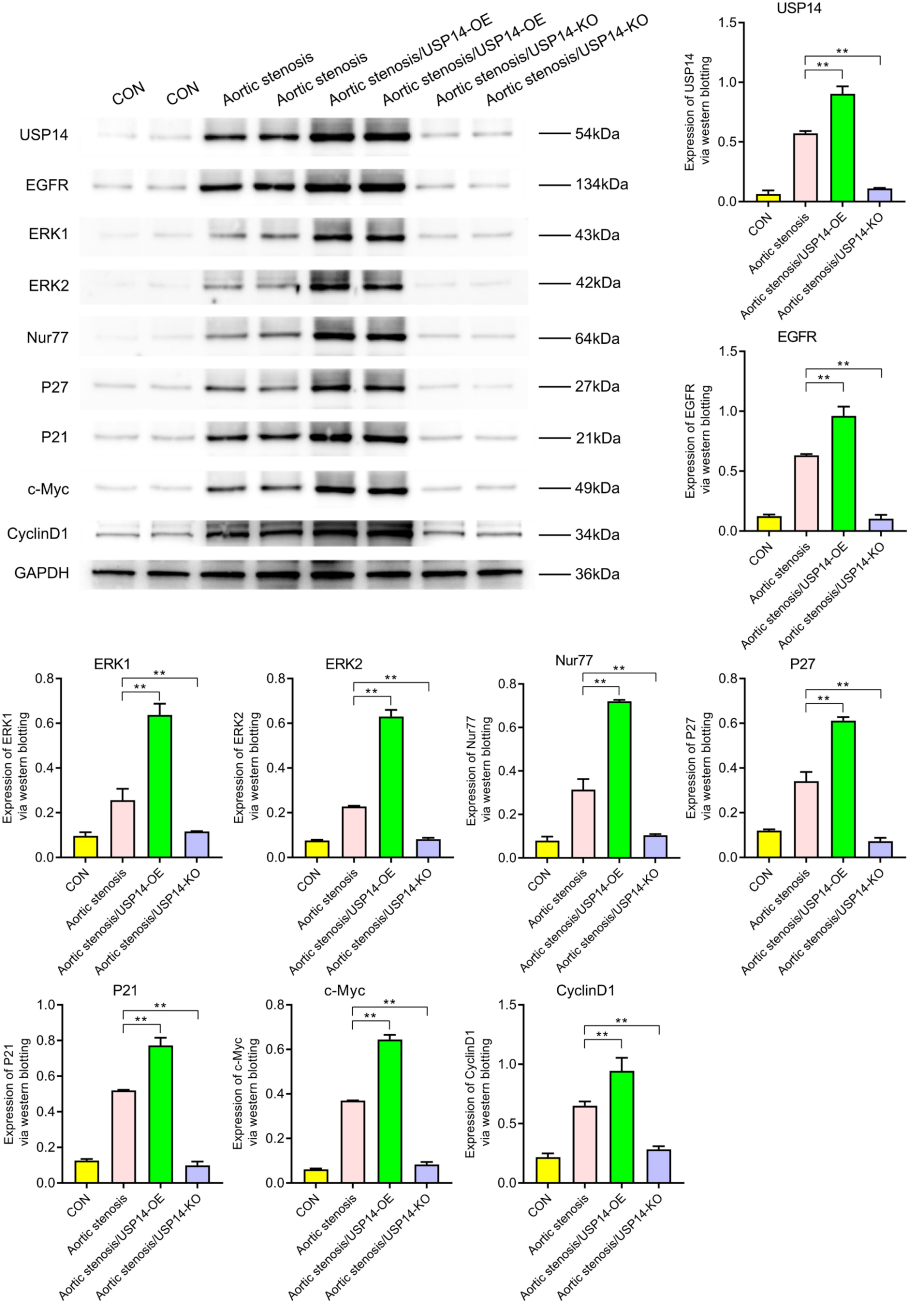
Western blot analysis of the effects of USP14 overexpression (USP14‐OE) and knockout (USP14‐KO) on the expression of cell proliferation and apoptosis‐related proteins EGFR, ERK1, ERK2, Nur77, P27, P21, c‐Myc and cyclin D1. Compared to the AS group, the USP14 overexpression group exhibited significant upregulation of these proteins, whereas the USP14 knockout group showed downregulation of these proteins.

**FIGURE 3:**
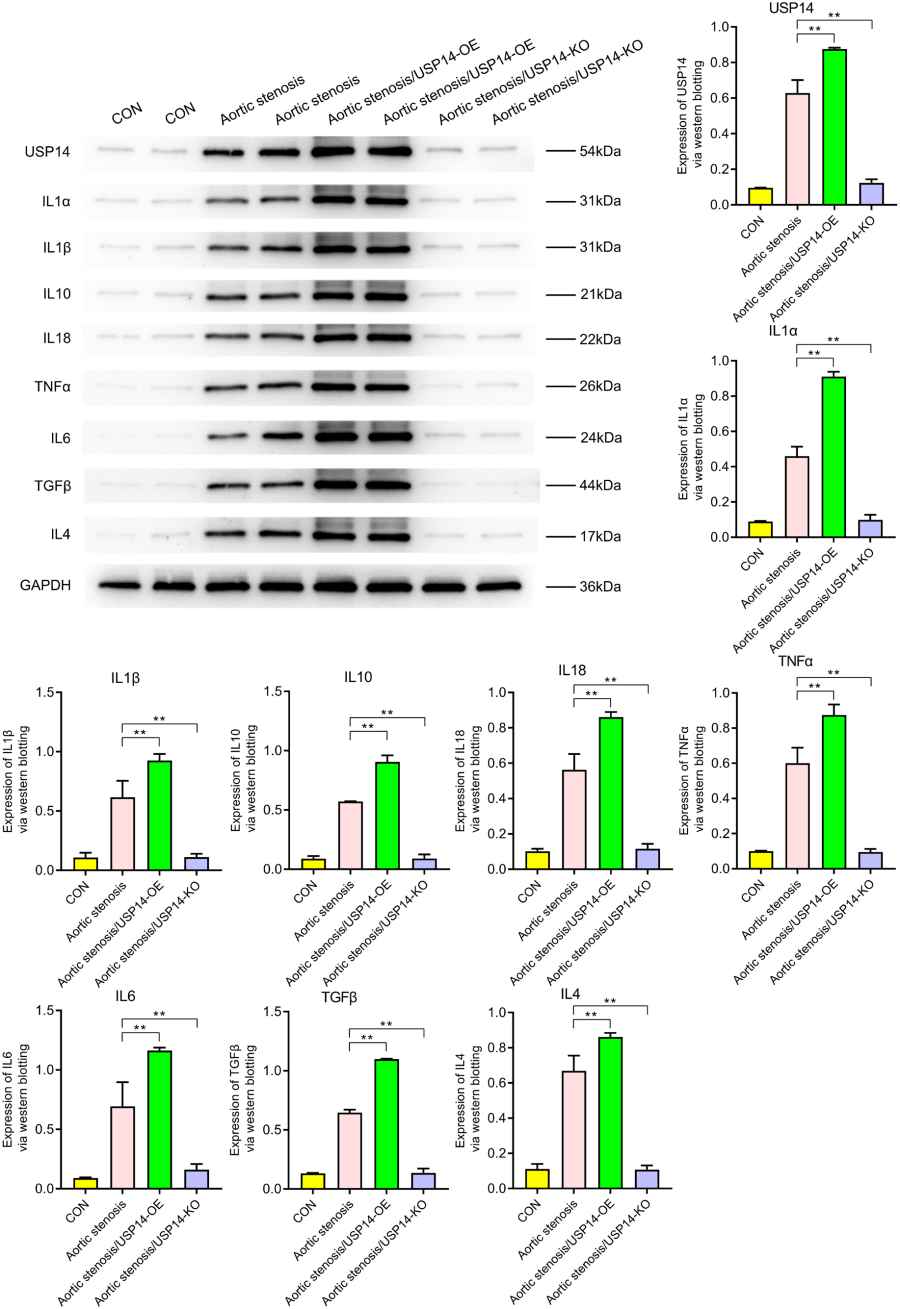
Western blot analysis of the effects of USP14 overexpression (USP14‐OE) and knockout (USP14‐KO) on the expression of various inflammatory factors IL1α, IL1β, IL10, IL18, TNFα, IL6, TGFβ and IL4. Compared to the AS group, the USP14 overexpression group exhibited significant upregulation of these inflammatory factors, whereas the USP14 knockout group showed downregulation of these factors.

**FIGURE 4:**
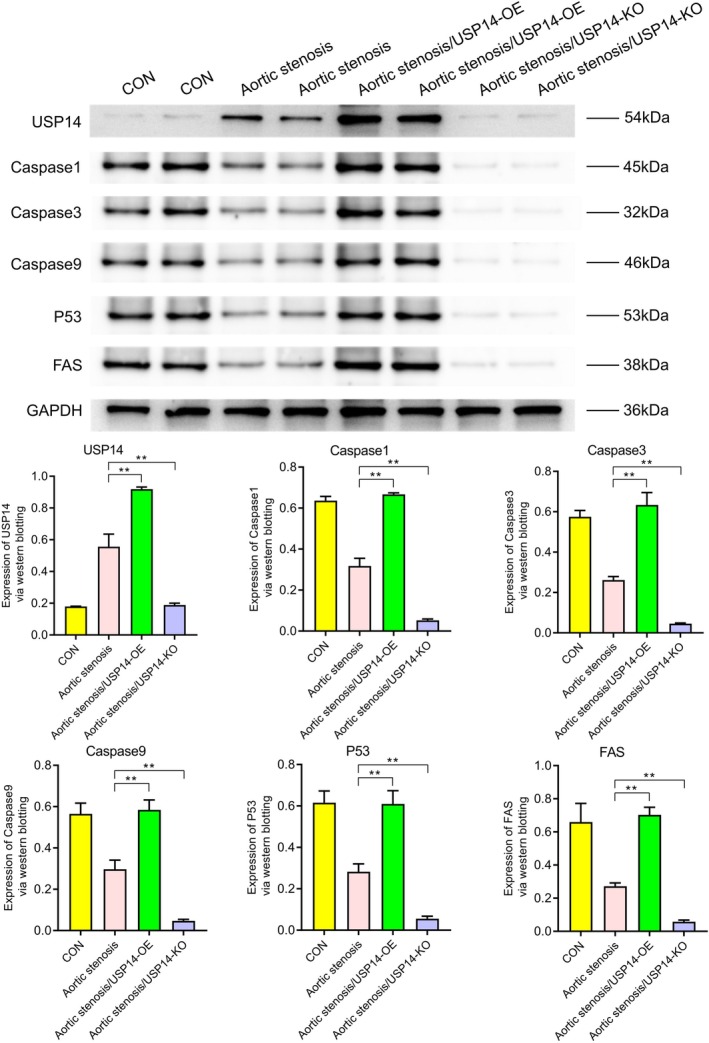
Western blot analysis of the effects of USP14 overexpression (USP14‐OE) and knockout (USP14‐KO) on the expression of apoptosis pathway‐related proteins Caspase1, Caspase3, Caspase9, P53 and FAS. Compared to the AS group, the USP14 overexpression group exhibited significant upregulation of these apoptosis proteins, whereas the USP14 knockout group showed downregulation of these proteins.

**FIGURE 5:**
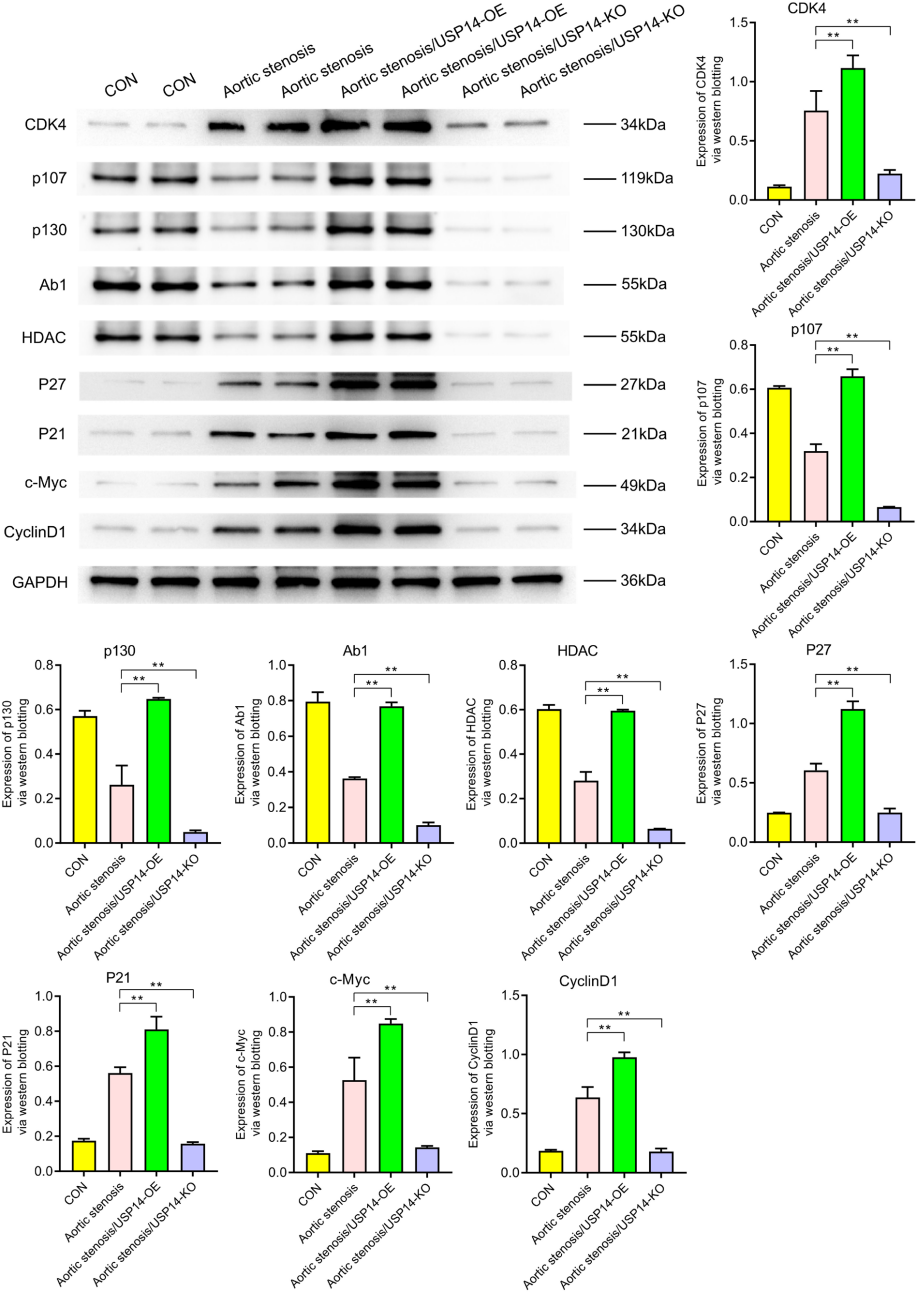
Western blot analysis of the effects of CDK4 overexpression (CDK4‐OE) and knockout (CDK4‐KO) on the expression of cell cycle‐related proteins CDK4, p107, p130, Ab1, HDAC, P27, P21, c‐Myc and cyclin D1. Compared to the AS group, the CDK4 overexpression group exhibited significant upregulation of these proteins, whereas the CDK4 knockout group showed downregulation of these proteins.

**FIGURE 6:**
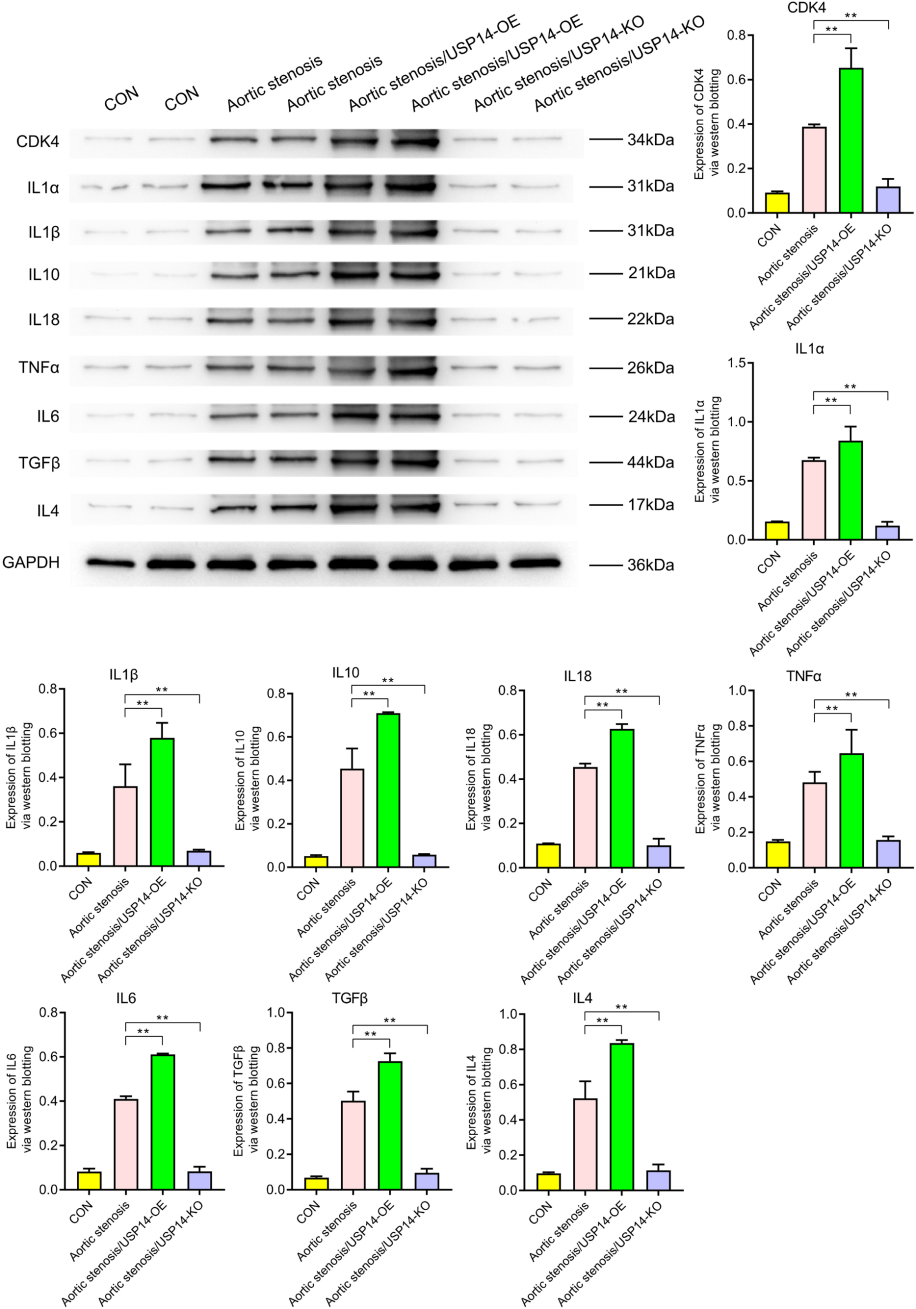
Western blot analysis of the effects of CDK4 overexpression (CDK4‐OE) and knockout (CDK4‐KO) on the expression of various inflammatory factors IL1α, IL1β, IL10, IL18, TNFα, IL6, TGFβ and IL4. Compared to the AS group, the CDK4 overexpression group exhibited significant upregulation of these inflammatory factors, whereas the CDK4 knockout group showed downregulation of these factors.

**FIGURE 7:**
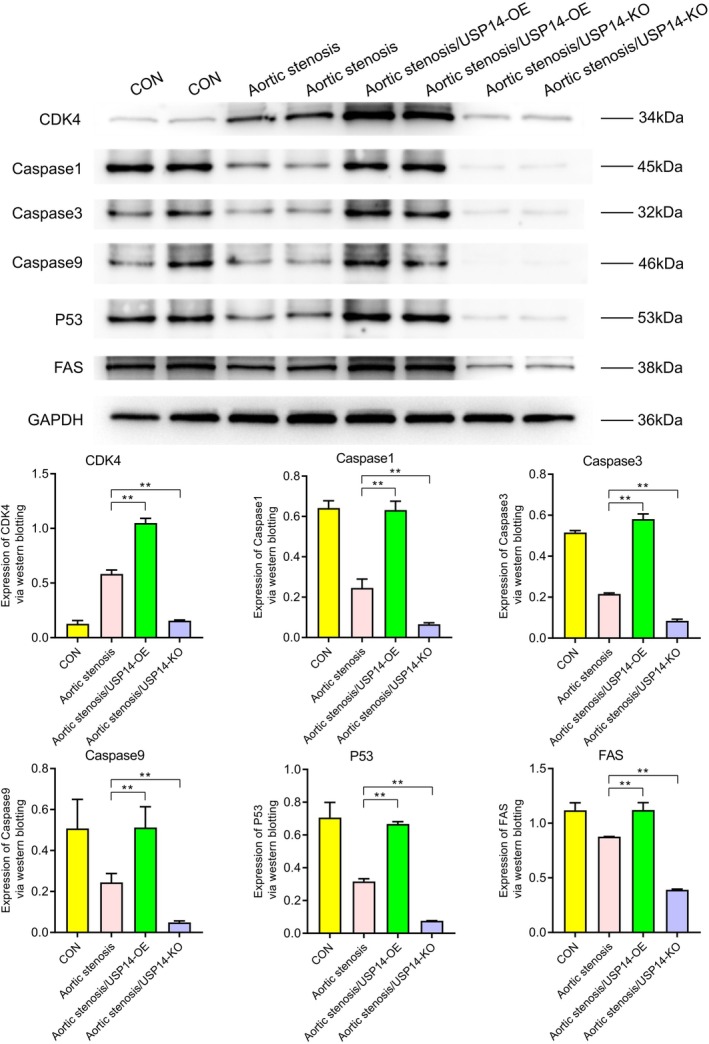
Western blot analysis of the effects of CDK4 overexpression (CDK4‐OE) and knockout (CDK4‐KO) on the expression of apoptosis pathway‐related proteins Caspase1, Caspase3, Caspase9, P53 and FAS. Compared to the AS group, the CDK4 overexpression group exhibited significant upregulation of these apoptosis proteins, whereas the CDK4 knockout group showed downregulation of these proteins.

Western blotting revealed the expression levels of various proteins under different conditions (CON, aortic stenosis, aortic stenosis/USP14‐OE, aortic stenosis/USP14‐KO). As shown in Figure 1, the expression of USP14 and CDK4 was significantly higher in aortic valve stenosis (AVS) tissues compared to control (CON) tissues. Regulation experiments on USP14 indicated that its overexpression significantly increased the expression levels of EGFR, ERK1, ERK2, Nur77, P27, P21, c‐Myc and cyclin D1, as shown in Figure 2. Additionally, Figures 3 and 4 demonstrated that USP14 overexpression also increased the expression of IL1α, IL1β, IL10, IL18, TNFα, IL6, TGFβ, IL4 and Caspase1, Caspase3, Caspase9, P53 and FAS. Conversely, knocking down USP14 resulted in a significant decrease in the expression of these genes. Similarly, overexpression of CDK4, as shown in Figures 5 and 6, led to the upregulation of CDK4, p107, p130, Ab1, HDAC, P27, P21, c‐Myc, cyclin D1, IL1α, IL1β, IL10, IL18, TNFα, IL6, TGFβ and IL4, whereas its knockout, as shown in Figure 7, led to the downregulation of these genes. These results enhance the understanding of the roles of USP14 and CDK4 in the pathophysiology of aortic stenosis and provide new targets and strategies for related disease treatments.

### 3.2 Co‐Immunoprecipitation and Western Blot Analysis of USP14 and CDK4

**FIGURE 8:**
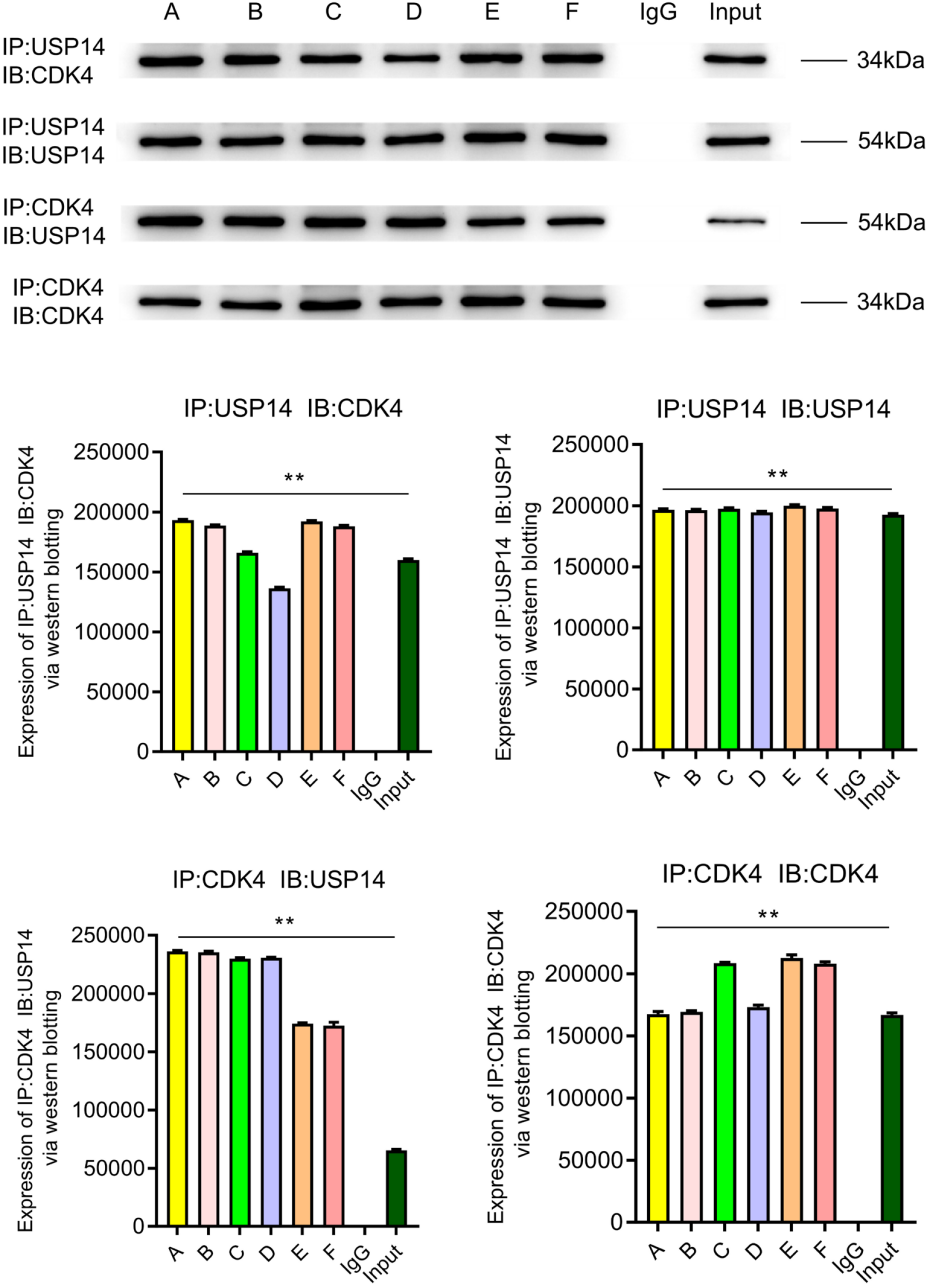
Western blot analysis of the co‐immunoprecipitation (Co‐IP) of USP14 and CDK4. The results indicate a significant interaction between USP14 and CDK4 in the AS model.

Figure 8 demonstrates a significant interaction between USP14 and CDK4 in AVS tissues. Co‐immunoprecipitation experiments indicated that when USP14 was immunoprecipitated, CDK4 could be significantly detected, and vice versa, suggesting a direct or indirect binding relationship at the molecular level. This result implies that USP14 and CDK4 might jointly participate in the development and progression of aortic valve stenosis, highlighting their important role in regulating the molecular mechanisms of this disease.

### 3.3 PCR Analysis

**FIGURE 9:**
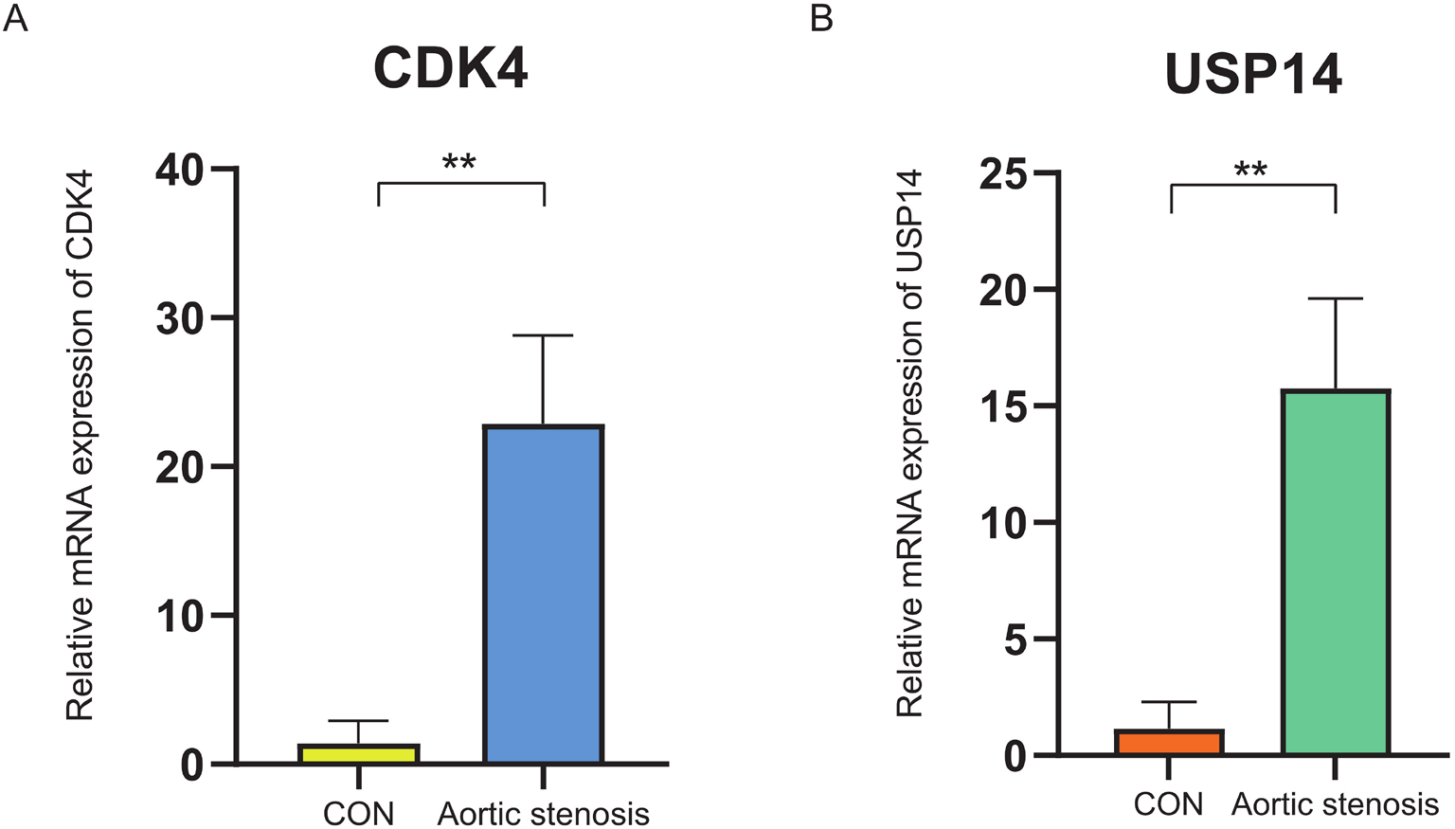
Quantitative RT‐PCR analysis of USP14 mRNA and CDK4 mRNA levels in AS and CON tissues. (A) The mRNA expression level of USP14 is significantly higher in the AS group compared to the CON group. (B) The mRNA expression level of CDK4 is significantly higher in the AS group compared to the CON group.

Quantitative RT‐PCR (qRT‐PCR) was performed to detect mRNA expression levels of USP14 and CDK4 in AVS and CON tissues. The results showed that USP14 mRNA levels were significantly higher in the AVS group compared to the CON group (Figure 9A), consistent with the protein expression data. Similarly, CDK4 mRNA levels were significantly higher in the AVS group compared to the CON group (Figure 9B).

### 3.4 HE Staining

**FIGURE 10:**
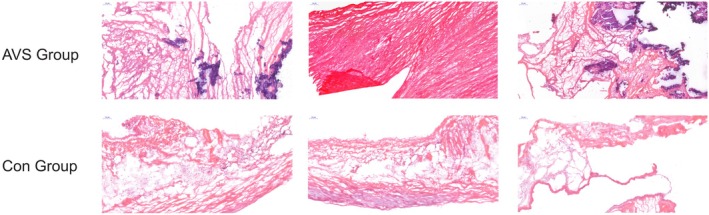
HE staining results of each group (× 100).

As shown in Figure 10, HE staining results indicated that in the AVS group, aortic valve tissues exhibited significant thickening and disordered arrangement. There was a notable accumulation of extracellular matrix and fibrosis. Nuclei were deeply stained, indicating significant cellular proliferation and inflammatory cell infiltration, suggesting strong pathological changes. In contrast, in the CON group, aortic valve tissues displayed normal structure without significant thickening or disorganisation. Cellular morphology was normal, extracellular matrix distribution was even and no significant fibrosis was observed. Nuclei were uniformly stained without noticeable inflammatory cell infiltration, indicating a healthy tissue state.

The HE staining results highlighted significant structural and cellular morphology changes in the aortic valve stenosis group compared to the control group. These changes included tissue thickening, fibrosis, cellular proliferation and inflammation infiltration, indicating severe pathological alterations in the AVS group. In contrast, the control group exhibited normal tissue structure and cell morphology without pathological changes. These findings support the critical roles of USP14 and CDK4 in the development and progression of aortic valve stenosis, further validating their potential as therapeutic targets.

### 3.5 Molecular Docking Analysis

#### 3.5.1 Screening

Molecular docking analysis of USP14 and CDK4 proteins provided binding energy and contact site information, along with the specific amino acid residues involved in binding (Table 1).

**TABLE 1:** The docking results of two target proteins.

Protein1	Protein2	Binding energy (kcal/mol)	Contact sites (Protein1)	Contact sites (Protein2)
USP14	CDK4	−324.29	THR‐299, ASN‐308, ARG‐209, GLU‐296, ARG‐293, GLU‐202, GLU‐145, GLU‐297	SER‐166, TYR‐167, ALA‐133, ARG‐55, ARG‐62, ARG‐288, SER‐52, ILE‐51

#### 3.5.2 Interaction Analysis of USP14 and CDK4

**FIGURE 11:**
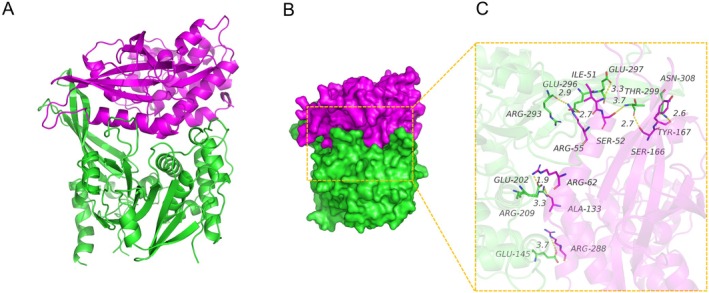
The binding mode of the complex USP14 with CDK4. (A) The backbone of protein was rendered in tube and coloured in green (USP14) and red (CDK4). (B) USP14 with CDK4 protein is rendered by the surface. (C) The detail binding mode of USP14 with CDK4. Yellow dash represents hydrogen bond or salt bridge.

The binding score between USP14 and CDK4 was −324.29 kcal/mol. The binding sites on USP14 included residues THR‐299, ASN‐308, ARG‐209, GLU‐296, ARG‐293, GLU‐202, GLU‐145 and GLU‐297, while the binding sites on CDK4 included residues SER‐166, TYR‐167, ALA‐133, ARG‐55, ARG‐62, ARG‐288, SER‐52 and ILE‐51. These contact residues formed various interactions such as salt bridges, hydrogen bonds and hydrophobic interactions, effectively enhancing the stability of the USP14 and CDK4 protein complex. Additionally, the surface matching of USP14 and CDK4 proteins facilitated the formation of a stable binding interaction (Figure 11).

These results elucidated the significant upregulation of USP14 and CDK4 in aortic stenosis tissues compared to non‐stenotic tissues. Knockdown and overexpression studies provided deeper insights into the mutual regulation mechanisms between USP14 and CDK4, suggesting that these proteins might synergistically influence the pathogenesis of aortic stenosis. Molecular docking analysis further supported the physical interaction between USP14 and CDK4, potentially underlying their combined effects on cellular signalling pathways.

## 4 Discussion

Aortic valve stenosis (AVS) is a prevalent heart valve disease characterised by the thickening and stiffening of aortic valve leaflets due to calcification and fibrosis, leading to valve orifice narrowing. This pathological state results in obstructed left ventricular ejection, increasing the load on the left ventricle, and ultimately causing left ventricular dysfunction, thereby raising the risk of adverse events [14, 15]. Epidemiological data indicate that the incidence of AVS rises significantly with age, particularly in individuals over 65, making it a major cause of cardiovascular‐related mortality in the elderly [16, 17]. The pathogenesis of AVS involves intricate molecular and cellular processes, including inflammatory responses, lipid deposition, calcification and fibrosis [18]. In the inflammatory response aspect, studies have shown that macrophages heavily infiltrate AVS lesion tissues, releasing various cytokines and chemokines that promote inflammation and calcification [19]. Moreover, lipid deposition plays a crucial role in the early stages of AVS, akin to the process of atherosclerosis, where low‐density lipoprotein accumulates beneath the aortic valve endothelium, gets oxidised and is subsequently ingested by macrophages, forming foam cells that further exacerbate inflammation [20]. Calcification and fibrosis are the main pathological features in the late stages of AVS. Research indicates that bone morphogenetic proteins and transforming growth factor‐beta signalling pathways are pivotal in the calcification process [21]. These signalling pathways activate osteoblast‐like differentiation, promoting calcium salt deposition and bone‐like matrix formation. In fibrosis, the activation and proliferation of fibroblasts and myofibroblasts are primary contributors. These cells secrete large amounts of extracellular matrix proteins, such as collagen, leading to thickening and stiffening of the valve [22].

Clinically, AVS manifests in various forms, including angina, dyspnoea and syncope. In severe cases, it can lead to heart failure, significantly affecting the patient's quality of life and prognosis [23]. Currently, surgical and transcatheter aortic valve replacement are the primary treatments for AVS. However, these methods are associated with high trauma, costs and complications, and they do not address the underlying cause of the disease [24, 25]. Therefore, understanding the molecular mechanisms of AVS is crucial for developing new therapeutic strategies and targeted drugs. Our study primarily shows that USP14 is highly expressed in stenotic aortic valve tissues.

USP14 is a deubiquitinating enzyme that plays a critical role in maintaining protein homeostasis and regulating various biological processes, including protein degradation, cell cycle, apoptosis and autophagy. USP14 belongs to the ubiquitin‐specific protease family, whose members regulate protein stability and function by removing ubiquitin chains from proteins [26]. USP1's primary function is to control the process of protein degradation, particularly by removing ubiquitin from proteins destined for degradation by the 26S proteasome, thereby affecting the efficiency of protein degradation [27]. Under normal physiological conditions, USP14 maintains cellular protein balance and homeostasis by regulating protein degradation. Its function is especially crucial in the nervous system, where USP14 regulates protein degradation, contributing to neuronal growth and function maintenance [28]. The absence or dysfunction of USP14 is associated with several neurodegenerative diseases, such as Alzheimer's and Parkinson's diseases, which are characterised by protein aggregation and neuronal damage [29]. In summary, as an important deubiquitinating enzyme, USP14 plays a key role in various physiological and pathological processes by regulating protein degradation. In‐depth research on USP1's functions and mechanisms not only helps to elucidate the pathogenesis of related diseases but also provides a scientific basis for developing new therapeutic strategies.

In recent years, research on USP14 has deepened, particularly its potential therapeutic target role in various diseases, attracting widespread attention. USP14 plays a crucial role in cancer research. Studies have shown that USP14 regulates tumour growth and progression by inhibiting deubiquitination activity in the 19S proteasome. Specifically, in p53‐deficient tumours, USP14 activity may lead to increased stability of cell cycle proteins such as cyclin E1, promoting tumour cell proliferation and inhibiting the degradation of apoptosis‐related proteins, enhancing tumour cell survival. Using specific USP14 inhibitors like b‐AP15 can effectively inhibit tumour growth and improve survival in tumour‐bearing mice by regulating cell cycle and apoptosis pathways, providing a potential treatment strategy for p53‐deficient tumours [30]. Other studies have found that USP14 stabilises BACH1 by deubiquitination, regulating heme metabolism through the NRF2 signalling pathway and promoting cancer cell invasion in ovarian cancer [31]. In non‐alcoholic fatty liver disease, USP14 stabilises HSP90AA1 through deubiquitination, leading to increased levels of CYP2E1 and further exacerbating disease progression [32]. Additionally, USP14 inhibits the degradation of IκBα, thereby suppressing NFκB signalling and enhancing sensitivity to TNFα‐induced cell death in head and neck squamous cell carcinoma, offering a potential strategy to overcome TNFα resistance in these cancers [33]. USP14's role in cardiovascular diseases is also gaining attention. Studies have found that USP14 expression is downregulated in atherosclerosis patients and endothelial cells stimulated by oxidised low‐density lipoprotein. Overexpression of USP14 can inhibit the activation of NF‐κB signalling and the production of adhesion molecules, primarily by regulating endothelial cell inflammatory activation through inhibition of NLRC5 degradation. Additionally, in vivo experiments confirmed USP14's inhibitory effect on atherosclerotic lesions, suggesting that USP14 may be a potential target for treating atherosclerosis [34].

In aortic valve stenosis, the mechanism of USP14's action is not yet fully understood. In the pathological process of aortic valve stenosis (AVS), the regulation of USP14 on cell proliferation, apoptosis and fibrosis may involve multi‐dimensional molecular mechanisms [35]. As a key deubiquitinating enzyme, USP14 can participate in the core pathological link of AVS by stabilising the expression level of specific substrate proteins. At the level of cell proliferation, it may target cell cycle regulatory proteins (such as cyclin D1) and inhibit their degradation by deubiquitinating modification, thereby promoting the abnormal proliferation of valvular interstitial cells. In the regulation of apoptosis, USP14 may act on apoptosis‐related proteins (such as Bcl‐2 family members) by maintaining the stability of anti‐apoptotic proteins or degrading pro‐apoptotic proteins, leading to the imbalance of apoptosis of valve cells and aggravating the damage of valve structure. In the process of fibrosis, USP14 may stabilise the key molecules in the transforming growth factor‐β (TGF‐β) signalling pathway (such as Smad3), enhance the transcriptional activity of its downstream fibrosis‐related genes (such as α‐SMA and collagen I) and promote the transformation of valve interstitial cells into myofibroblasts and the excessive deposition of extracellular matrix. The synergistic effect of the above mechanisms may jointly promote the progression of valve calcification and fibrosis in AVS.

One study confirmed through gene expression analysis and molecular experiments that USP14's expression level is significantly elevated in samples from patients with aortic valve stenosis. This finding suggests that USP14 may be involved in the development and progression of aortic valve stenosis, potentially serving as a molecular target for early diagnosis and specific treatment of the disease [36]. Our research results support the critical role of USP14 in AVS. HE staining results showed significant thickening, fibrosis and extracellular matrix accumulation in aortic valve tissues in the AVS group, whereas the control group had normal tissue structure. Western blot experiments further validated the interaction between USP14 and CDK4, indicating that USP14 may be involved in the pathological process of AVS by regulating CDK4 expression and function. These results suggest that USP14 plays a key role in the development and progression of aortic valve stenosis and may become a new target for AVS treatment. Our research results show that the expression level of USP14 in the aortic valve tissue of AVS patients is significantly higher than that in the normal control group. This finding suggests that USP14 may play an important role in the pathological process of AVS. Through cell experiments, we found that knocking out the USP14 gene can significantly inhibit the proliferation and migration abilities of aortic valve interstitial cells, further supporting the critical role of USP14 in AVS. Additionally, the interaction between USP14 and the cell cycle protein CDK4 is another significant finding of this study. CDK4 plays an essential role in cell proliferation and differentiation, and our research indicates that USP14 can influence the function of aortic valve interstitial cells by regulating CDK4 expression levels. This discovery provides a new perspective for understanding the mechanism of USP14's role in AVS.

### 4.1 Limitations

Despite revealing the important role of USP14 in AVS, this study has some limitations. It mainly relies on in vitro cell experiments and lacks validation in in vivo animal models. Moreover, the interaction mechanisms between USP14 and other signalling pathways and effector molecules require further investigation. Future studies should validate the function of USP14 in animal models and explore its potential in AVS treatment.

## 5 Conclusion

In summary, this study reveals the critical role of USP14 in aortic valve stenosis through bioinformatics and experimental validation. USP14 may participate in the occurrence and development of AVS by regulating cell proliferation, apoptosis and fibrosis processes. Future research should further validate these findings and explore the potential of USP14 as a therapeutic target, providing new treatment strategies for AVS patients.

## Author Contributions

**Yin Yang:** conceptualization (equal), methodology (equal), writing – review and editing (equal). **Bo‐Chen Yao:** data curation (equal), formal analysis (equal), methodology (equal), writing – original draft (equal). **Jing‐Hui Li:** data curation (equal), methodology (equal), writing – review and editing (equal). **Qing‐Liang Chen:** formal analysis (equal), investigation (equal), writing – original draft (equal). **Nan Jiang:** data curation (equal), formal analysis (equal), software (equal), writing – original draft (equal). **Lian‐Qun Wang:** data curation (equal), formal analysis (equal), writing – original draft (equal). **Zhi‐Gang Guo:** data curation (equal), formal analysis (equal), writing – original draft (equal).

## Ethics Statement

This study was approved by the ethics committee of Chest Hospital, Tianjin University and Tianjin Chest Hospital.

## Conflicts of Interest

The authors declare no conflicts of interest.

## Data Availability

The datasets generated during and/or analysed during the current study are available from the corresponding author on reasonable request.
